# Phase I study of intra-osseous co-transplantation of a single-unit cord blood and mesenchymal stromal cells with reduced intensity conditioning regimens

**DOI:** 10.3389/fonc.2023.1186532

**Published:** 2023-05-03

**Authors:** Jiasheng Wang, Farhad Sanati, Amin Firoozmand, Pingfu Fu, Merle Kolk, Jane Reese-Koc, Marcos de Lima, Kirsten Boughan, Brenda Cooper, Paolo Caimi, Molly Gallogly, Folashade Otegbeye, Benjamin Tomlinson, Leland Metheny

**Affiliations:** ^1^ Department of Hematology/Oncology, University Hospitals Seidman Cancer Center, Cleveland, OH, United States; ^2^ Case Western Reserve University, Cleveland, OH, United States; ^3^ Adult Hematologic Malignancies & Stem Cell Transplantation Service, University Hospitals Seidman Cancer Center, Cleveland, OH, United States; ^4^ Division of Hematology, Ohio State University, Columbus, OH, United States; ^5^ Department of Hematology/Oncology, Louis Stokes Veterans Affairs Medical Center, Cleveland, OH, United States; ^6^ Blood and Marrow Transplant, Cleveland Clinic Foundation, Cleveland, OH, United States; ^7^ Clinical Research Division, Fred Hutchison Cancer Center, Seattle, WA, United States

**Keywords:** intra-osseous, cord blood, mesenchymal stromal cells, allogeneic hematopoietic cell transplantation, reduced intensity conditioning

## Abstract

Cord blood (CB) is a valuable graft source for patients undergoing allogeneic hematopoietic cell transplant (HCT) who lack human leukocyte antigen (HLA)-matched donors. However, single-unit CB-HCT is limited by the insufficient cell dose and slow engraftment. To overcome these limitations, we combined a single-unit CB with third-party healthy donors’ bone marrow (BM) derived mesenchymal stromal cells (MSCs) to improve engraftment and injected intra-osseously (IO) to enhance homing. In this phase I clinical trial, six patients with high-risk hematologic malignancies were enrolled and received allogeneic HCT using reduced intensity conditioning regimens. The primary objective was to determine the engraftment rate at day 42. The median age of enrolled patients was 68 years, and only one patient was in complete remission at the time of HCT. The median CB total nucleated cell dose was 3.2x10^7^/kg. No serious adverse events were reported. Two patients had early deaths due to persistent disease and multi-drug resistant bacterial infection, respectively. Of the remaining four evaluable patients, all had successful neutrophil engraftment in a median of 17.5 days. No grade 3 or higher acute graft-versus-host disease (GvHD) was observed, and only one patient developed moderate-extensive chronic GvHD. In conclusion, IO co-transplantation of a single-unit CB and MSCs was feasible and resulted in a reasonable engraftment rate in these very high-risk patients.

## Introduction

Cord blood (CB) has been established as an important graft source for allogeneic hematopoietic cell transplantation (allo-HCT). CB hematopoietic stem cells possess a higher capability for proliferation than bone marrow stem cells (1). Moreover, the immune tolerant state and low frequency of alloreactive T cells in CB have led to a lower incidence of graft-versus-host disease (GVHD) than BM-based allo-HCT. Additionally, CB requires less restrictive HLA matching for successful engraftment (2, 3). However, the limited number of stem cells in a single CB unit and slow engraftment remain significant challenges in CB-HCT, leading to high rates of infection and transplant-related mortality (TRM) (4). Therefore, there is a need for further development of techniques to improve CB engraftment.

In 2008, Frassoni et al. first demonstrated that directly injecting CB stem cells into the bone marrow (intra-osseous or IO injection) could improve CB homing to the BM, by potentially reducing trapping in solid organs like the liver after intravenous injection (5). Retrospective studies showed that IO injection of a single-unit CB was associated with an improved neutrophil engraftment compared to double-unit CB-HCT, despite a lower cell dose (6). Additionally, co-transplantation of CB with mesenchymal stromal cells (MSCs) have been explored in pre-clinical and early clinical studies to facilitate CB engraftment (7). As a part of the BM microenvironment, MSCs play a key role in regulating and maintaining hematopoietic stem cells. Moreover, given their immunomodulatory effects, co-transplantation with MSCs may also decrease the risk of graft-versus-host disease (GvHD). In a study by Lee et al., co-transplantation of third-party MSCs with single- or double-unit CB in patients with hematologic malignancies was safe and associated with improved engraftment compared to historic controls (8).

Preclinical study from our group showed that combining the above two approaches – IO injection and co-transplantation of CB and MSC – had resulted in significantly improved engraftment in mice compared to IV infusion or IO injection alone (9). Based on these findings and previously demonstrated safety of third-party MSCs, we conducted a phase I, single arm study of IO co-transplantation of single-unit CB and healthy donor-derived BM MSCs using reduced intensity conditioning (RIC) regimens in patients with high-risk hematological malignancies who lacked an available HLA matched related, unrelated, or haploidentical donor.

## Methods

### Patients

From 7/2015 to 12/2019, adult patients (≤75 years old) with a diagnosis of high-risk hematological malignancies who were eligible for reduced-intensity conditioning (RIC) regimen allogeneic hematopoietic cell transplantation and did not have HLA matched related, unrelated, or haploidentical donors were screened for the trial enrollment. High-risk hematological malignancies include relapsed/refractory (R/R) acute myelogenous leukemia (AML) or newly diagnosed (ND) AML with adverse-risk cytogenetics, higher-risk myelodysplastic syndromes (MDS), or primary myelofibrosis (PMF). Patients are required to have cord blood units that were HLA matched at ≥4/6 (HLA-A, B, DRB1) with a minimum total nucleated cell (TNC) dose of 1.9x10^7^/kg according to our institutional guidelines. All subjects provided written informed consent.

### Study design

The primary objective of the study was to determine the feasibility of IO co-transplantation of a single-unit CB and MSCs with a RIC regimen. The feasibility was determined by the rate of primary graft failure less than 10%, which is based on historical data in single-unit CB-HCT where the rate of primary graft failure ranges between 10 to 20% (10). Primary engraftment failure was defined as absolute neutrophil count (ANC) <500/µl at day 42 without disease, or BM cellularity <10% at day 42 without evidence of donor engraftment, or hematopoietic recovery with donor chimerism <10% at day 42 or <40% at day 100. The chimerism was tested on the peripheral blood or bone marrow using a panel of 16 polymorphic DNA markers. ANC engraftment is defined as the first of three consecutive days with an ANC ≥500 cells/µL. Platelet engraftment is defined as the first of seven consecutive days with a count ≥20,000 cells/µL without transfusion support. The study was approved by the Case Comprehensive Cancer Center Institution Review Board and was conducted in accordance with the Declaration of Helsinki. The study was registered at clinicaltrials.gov (NCT02181478).

### Preparation of BM-derived MSCs

MSCs were derived from the bone marrow of healthy individuals who were not HLA matched to the potential recipients. They were tested negative for hepatitis B surface antigen and core antibody, hepatitis C antibodies and PCR, HIV antibodies and PCR, HTLV antibodies, treponema pallidum antibodies, CMV antibodies, or trypanosoma cruzi antibodies. All subjects provided written informed consent. MSCs were produced under the guidelines set forth by the Foundation for the Accreditation of Cellular Therapy (FACT) in the Clinical Research Unit at University Hospitals. Briefly, after a 1.073 gm/ml Percoll gradient centrifugation, mononuclear cell layer was removed and placed into tissue flasks containing MSC culture medium with DMEM-low glucose (Arteriocyte Medical Systems, Inc). The culture medium was replaced every 3 to 4 days and the cells were monitored under a microscope. The primary culture was harvested at approximately 10 to 16 days and then cryopreserved. For culture expansion, vials of cryopreserved MCSs were thawed and cultured for additional 2 to 3 weeks. Once release testing (sterility, mycoplasma, endotoxin, and surface marker analysis) were passed, cells were released to the principal investigators either fresh or cryopreserved. A total dosage of MSCs of 2x10^6^ cells/kg (+/-20%) was targeted based on our previous experience (11).

### Intra-osseous co-transplant of MSCs and CB

The RIC regimens included cyclophosphamide 50 mg/kg on day -6, fludarabine 40 mg/m^2^ daily on days -6 to -2, and total body irradiation 200 cGy on day -1(Flu/Cy/TBI); or fludarabine 40 mg/m^2^ daily on days -5 to -2, melphalan 100 or 140 mg/m^2^ on day -2, and rabbit antithymocyte globulin (ATG) 1.25mg/kg on day -3 and 1.75mg/kg on day -2 (Flu/Mel/ATG). GVHD prophylaxis with mycophenolate mofetil (MMF) and cyclosporine were used with Flu/Cy/TBI conditioning, and MMF and tacrolimus were used with Flu/Mel/ATG conditioning. On the day of transplant (T0), a Jamshidi bone marrow needle was inserted into the posterior iliac crest of the patient after local anesthesia. An aspiration of about 2 mL was drawn to assess that the needle was securely inserted into the bone-marrow cavity. Subsequently, 25 mL of MSCs was infused over 5 minutes followed by 10 mL of CB suspension over 5 minutes. The process was then repeated on the contralateral posterior iliac crest using the remaining MSCs and CB.

### Statistical analysis

The overall survival (OS) was measured from the date of transplantation to the date of death and was censored at the date of last follow-up for survivors. The disease-free survival (DFS) was measured from the date of transplantation to the disease progression or the date of death and was censored at the date of last follow-up for those alive without disease progression. The cumulative incidence of platelet engraftment and neutrophil engraftment and survivor distribution was estimated using Kaplan-Meier methods and differences of engraftment (neutrophil and platelet) and survival (OS, DFS) between/among groups was examined by the log-rank test. All tests were two-sided and a p-value ≤ 0.05 was considered statistically significant. Statistical calculations and figures were created using R statistical software version 4.2.1 (R Foundation for Statistical Computing, Vienna, Austria).

## Results

### Patient characteristics

Between 2016 and 2019, 6 patients with high-risk hematologic malignancies received IO co-transplantation of CB and MSCs at the University Hospitals Cleveland Medical Center. The patients’ characteristics are summarized in **Table 1**. Three patients had AML; one relapsed after a previous allogeneic HCT and was transplanted with active disease, one with treatment-related AML who was transplanted with active disease, and one with adverse-risk cytogenetics who was transplanted at first complete remission. Two patients had higher-risk MDS; one was treatment related and the other was evolved from a myeloproliferative neoplasm. The remaining patient had PMF.

**Table 1:** Patients’ characteristics.

Characteristics	Patient number (%), N=6
Age, median (range)	68 (57-71)
Sex	
Male	3 (50)
Female	3 (50)
Race	
White	3 (50)
Black	2 (33)
Asian	1 (17)
Diagnosis	
Acute myeloid leukemia	3 (50)
Myelodysplastic syndrome	2 (33)
Primary myelofibrosis	1 (17)
Disease status at transplantation	
CR1	1 (17)
Active disease	2 (33)
Stable disease	3 (50)
HCT-CI	
1 to 2	3 (50)
3 or more	3 (50)
CB TNC dose, median (range), x10^7^/kg	3.2 (1.9-7.1)
CB CD34+ dose, median (range), x10^6^/kg	0.12 (0.03-0.37)
Conditioning regimen	
Flu/Cy/TBI	4 (67)
Flu/Mel/ATG	2 (33)
Karnofsky performance status, %	
70	3 (50)
80	3 (50)
HLA match	
5/6	4 (67)
6/6	2 (33)
GvHD prophylaxis	
MMF + Cyclosporin	4 (67)
MMF + Tacrolimus	2 (33)

CR, complete remission; HCT-CI, hematopoietic cell transplantation-specific comorbidity index; CB, cord blood; TNC, total nucleated cell; Flu, fludarabine; Cy, cyclophosphamide; TBI, total body irradiation; Mel, melphalan; ATG, antithymocyte globulin; GvHD, graft-versus-host disease; MMF, mycophenolate mofetil.

The median TNC in a single unit CB was 3.2x10^7^/kg (range 1.9 to 7.1x10^7^/kg) and the median CD34+ count was 0.12x10^6^/kg (range 0.03 to 0.37x10^6^/kg). Four patients received Flu/Cy/TBI conditioning with MMF and cyclosporin GvHD prophylaxis; the other two patients received Flu/Mel/ATG conditioning with MMF and tacrolimus GvHD prophylaxis.

### Engraftment

**Figure 1:**
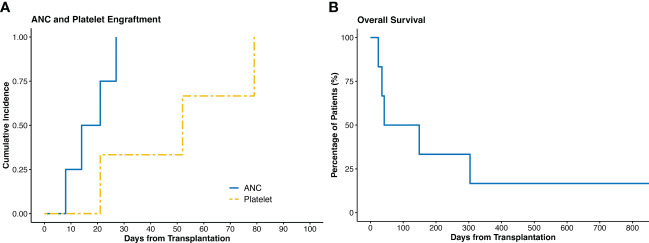
**(A)** Cumulative incidence of ANC and platelet engraftment. **(B)** Overall survival of all the patients. ANC, absolute neutrophil count.

The IO co-infusion was well tolerated in all patients. Of the four evaluable patients, all (100%) had successful engraftment. The median time to ANC and platelet engraftment was 17.5 (range 8 to 27) days and 52 (range 21 to 79) days, respectively. (**Figure 1A**)

### Survival outcomes

**Figure 2:**
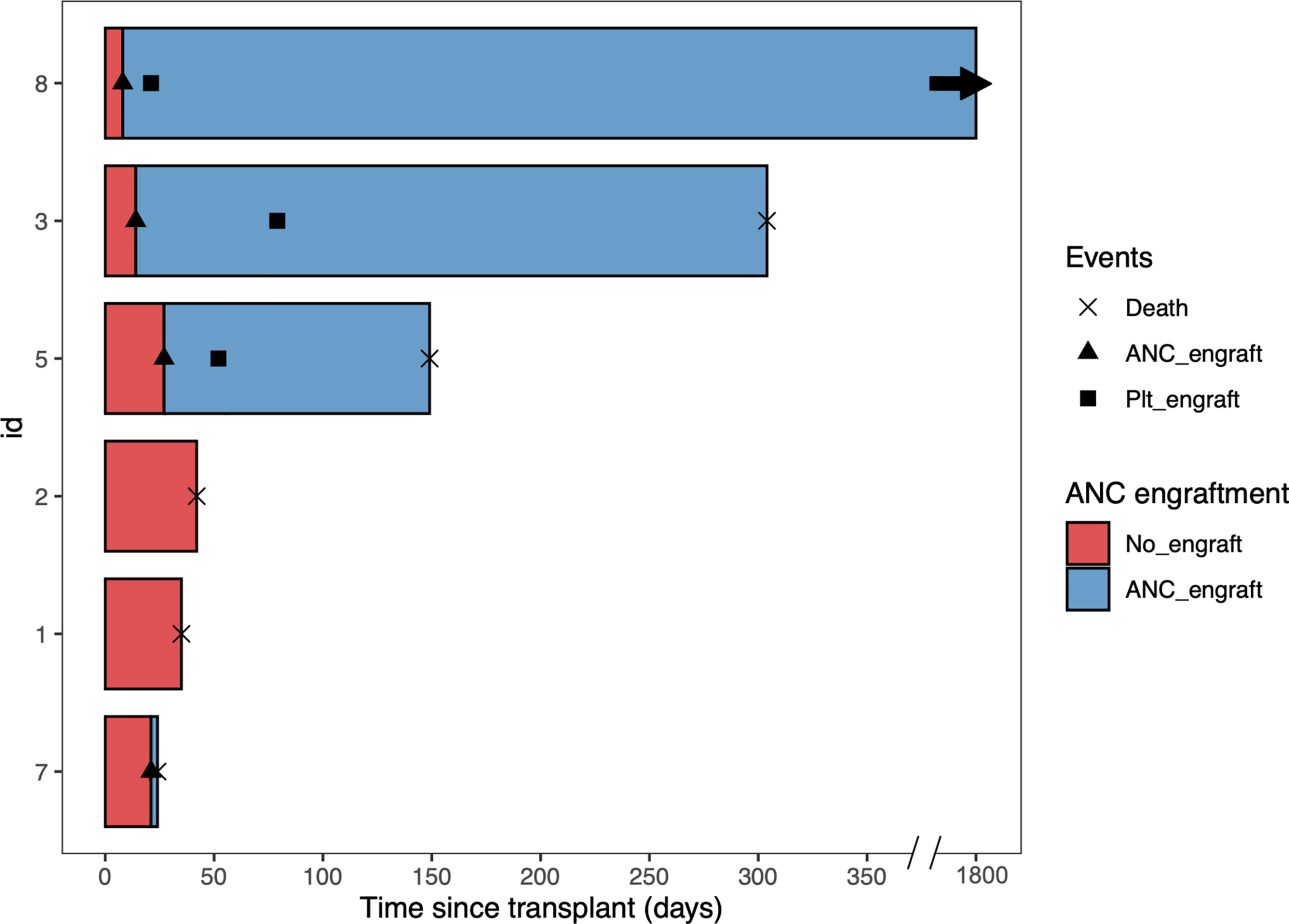
Swimmer plot of all the patients. Patient 1, 2, 3, and 8 received conditioning regimen of fludarabine, cyclophosphamide, and total body irradiation, while Patient 5 and 7 received conditioning regimen of fludarabine, melphalan, and rabbit antithymocyte globulin. ANC, absolute neutrophil count. Plt, platelet.

Three patients had early deaths within the first two months. One patient died at day 24 due to multiple cyclosporin-related complications, including posterior reversible encephalopathy syndrome (PRES), acute renal failure, and transplant-associated thrombotic microangiopathy, despite achieving ANC engraftment at day 21. The second patient with PMF died at day 35 due to multidrug resistant *Stenotrophomonas maltophilia* pneumonia. The third patient died at day 42 due to persistent disease – this patient had a previous allogeneic HCT and was transplanted with active disease. Of the remaining three patients, none had disease relapse. After a median follow up of 3.1 (range 0.8 to 59.8) months, the OS was 95.5 days. (**Figures 1B**, **2**)

### Acute and chronic GvHD

Altogether three patients develop acute GvHD; one and two patients developed overall grade 1 and grade 2 acute GvHD, respectively. One patient developed moderate-extensive chronic GvHD which resolved after corticosteroids.

## Discussion

In this phase I study, we enrolled six patients with high-risk hematologic malignancies who lacked HLA matched or haploidentical donors. Our approach of intra-osseous co-transplantation of single-unit CB and healthy donor-derived MSCs was feasible and led to a 100% engraftment rate in evaluable patients with a median ANC engraftment time of 17.5 days.

Insufficient cell dose is the most common restricting factor for CB-HCT (12). IO transplantation was developed to hypothetically reduce the required total stem cell dose by minimizing cell trapping in other organs. Multiple phase I/II studies have confirmed the feasibility of this approach, with engraftment rates around 80% and ANC engraftment time ranging from 17 to 23 days, despite lower than standard TNC and CD34+ cell doses (13–17). However, no randomized studies have confirmed its superiority over the standard intravenous administration. Another obstacle to CB-HCT is the slow engraftment, which may have contributed to the higher infection rate and transplant-related mortality when compared to alternative donor sources (18). Because MSCs are supporting cells for hematopoietic stem cells, co-infusing MSCs with CB has the potential to facilitate CB engraftment. This approach has been tested in early phase clinical trials in patients with hematologic malignancies as well as non-malignant disorders (7). Among trials studying MSC combined with CB in patients with hematologic malignancies, the doses of MSC range from 1 to 7x10^6^ cells/kg, leading to median ANC engraftment time from 12 to 19 days (8, 19). Moreover, low rates of acute GvHD were observed (8, 19). To our knowledge, however, trials combining the above two approaches have not been reported. In our study, all patients received single-unit CB, and four patients received CD34+ cell doses lower than 0.15x10^6^ cells/kg, which was the minimal dose recommended by the American Society of Transplantation and Cellular Therapy (ASTCT) (12). We decided to administer MSCs before CB to prevent CB from being flushed into the systemic circulation due to the larger volume of MSCs. Although this study is limited by a small sample size, all evaluable patients achieved engraftment with a median ANC engraftment time of 17.5 days, which is similar to the aforementioned studies. Moreover, two patients demonstrated rapid ANC recovery at day 8 and 14.

The MSCs used in our study were obtained from a limited number of unrelated healthy donors and were not HLA-matched with the recipients. This is because research has demonstrated that MSCs possess low immunogenicity and HCT recipients are known to exhibit tolerance towards HLA-mismatched MSCs (20). In fact, the sole MSC product that is currently seeking approval from the US Food and Drug Administration (FDA), remestemcel-L, is derived from HLA-unmatched donors.

We enrolled patients with very high-risk hematologic malignancies, including two with AML who underwent transplantation with active diseases. In a large retrospective study by the European society for Blood and Marrow Transplantation (EBMT) focusing on patients with active diseases at transplantation, traditional CB-HCT had a high rate of primary engraftment failure of more than 25% and an inferior overall survival compared to unrelated donors (21). However, in our study, patients lacked identifiable HLA-matched or haploidentical donors, making CB the only donor source for allogeneic HCT. Moreover, two patients received ATG as part of the conditioning regimen, which has been linked to increased early mortality due to delayed T-cell recovery and is no longer recommended for inclusion in conditioning (22). Nonetheless, these factors likely contributed to the unfavorable survival outcomes in our study, which was evidenced by three early deaths in the trial. However, of the remaining three patients who survived the early post-HCT period, all achieved good graft function, and none had disease relapse. Furthermore, a long-term survivor is off all immunosuppressive medications. Therefore, IO co-transplantation with MSC is worth further investigation in patients with lower risk diseases who lack an alternative donor and only a single-unit CB can be identified.

Additionally, co-transplantation with MSCs may reduce acute GvHD due to their immunomodulatory effects. In a seminal study by Le Blanc et al., BM-derived MSCs induced remission in a patient with grade IV treatment-resistant acute GvHD (23). In our study, one patient developed grade 1 and two patients developed grade 2 acute GvHD. Despite four patietns receiving 5/6 matched CB, no high-grade acute GvHD was observed. However, given the small sample size, no conclusions can be drawn.

The study is limited by the small patient size. Additionally, we used a single dose level of MSCs, and the impact of difference MSC doses on engraftment remains to be determined. Larger studies enrolling patients under complete remission or with only measurable residual diseases at the time of transplantation are necessary to further demonstrate the safety and efficacy of this approach.

## Conclusion

In conclusion, intra-osseous co-transplantation of single-unit CB and healthy donors’ BM-derived MSCs is feasible in patients with high-risk hematologic malignancies.

## Data availability statement

The raw data supporting the conclusions of this article will be made available by the authors, without undue reservation.

## Ethics statement

The studies involving human participants were reviewed and approved by Case Western Reserve University IRB. The patients/participants provided their written informed consent to participate in this study.

## Author contributions

Concept and design: LM, ML Data collection and analysis: JW, PF, MK Manuscript writing: JW, LM Final approval of manuscript: All authors.

## References

[1] Wang JC Doedens M Dick JE. Primitive human hematopoietic cells are enriched in cord blood compared with adult bone marrow or mobilized peripheral blood as measured by the quantitative *in vivo* SCID-repopulating cell assay. Blood (1997) 89(11):3919–24. doi: 10.1182/blood.V89.11.3919 9166828

[2] Rocha V Wagner JE Jr. Sobocinski KA Klein JP Zhang MJ Horowitz MM. Graft-versus-host disease in children who have received a cord-blood or bone marrow transplant from an HLA-identical sibling. eurocord and international bone marrow transplant registry working committee on alternative donor and stem cell sources. N Engl J Med (2000) 342(25):1846–54. doi: 10.1056/NEJM200006223422501 10861319

[3] Eapen M Rubinstein P Zhang MJ Stevens C Kurtzberg J Scaradavou A. Outcomes of transplantation of unrelated donor umbilical cord blood and bone marrow in children with acute leukaemia: a comparison study. Lancet (2007) 369(9577):1947–54. doi: 10.1016/S0140-6736(07)60915-5 17560447

[4] Weisdorf D Eapen M Ruggeri A Zhang MJ Zhong X Brunstein C. Alternative donor transplantation for older patients with acute myeloid leukemia in first complete remission: a center for international blood and marrow transplant research-eurocord analysis. Biol Blood Marrow Transplant. (2014) 20(6):816–22. doi: 10.1016/j.bbmt.2014.02.020 PMC4085692 24582782

[5] Frassoni F Gualandi F Podesta M Raiola AM Ibatici A Piaggio G. Direct intrabone transplant of unrelated cord-blood cells in acute leukaemia: a phase I/II study. Lancet Oncol (2008) 9(9):831–9. doi: 10.1016/S1470-2045(08)70180-3 18693069

[6] Rocha V Labopin M Ruggeri A Podesta M Gallamini A Bonifazi F. Unrelated cord blood transplantation: outcomes after single-unit intrabone injection compared with double-unit intravenous injection in patients with hematological malignancies. Transplantation (2013) 95(10):1284–91. doi: 10.1097/TP.0b013e318288ca4d 23507699

[7] Li T Luo C Zhang J Wei L Sun W Xie Q. Efficacy and safety of mesenchymal stem cells co-infusion in allogeneic hematopoietic stem cell transplantation: a systematic review and meta-analysis. Stem Cell Res Ther (2021) 12(1):246. doi: 10.1186/s13287-020-02064-0 33879242 PMC8056684

[8] Lee SH Lee MW Yoo KH Kim DS Son MH Sung KW. Co-Transplantation of third-party umbilical cord blood-derived MSCs promotes engraftment in children undergoing unrelated umbilical cord blood transplantation. Bone Marrow Transplant. (2013) 48(8):1040–5. doi: 10.1038/bmt.2013.7 23396407

[9] Metheny L 3rd Eid S Lingas K Reese J Meyerson H Tong A. Intra-osseous Co-transplantation of CD34-selected umbilical cord blood and mesenchymal stromal cells. Hematol Med Oncol (2016) 1(1):25–9. doi: 10.15761/HMO.1000105 PMC5117423 27882356

[10] Barker JN Scaradavou A Stevens CE. Combined effect of total nucleated cell dose and HLA match on transplantation outcome in 1061 cord blood recipients with hematologic malignancies. Blood (2010) 115(9):1843–9. doi: 10.1182/blood-2009-07-231068 PMC5003507 20029048

[11] Lazarus HM Koc ON Devine SM Curtin P Maziarz RT Holland HK. Cotransplantation of HLA-identical sibling culture-expanded mesenchymal stem cells and hematopoietic stem cells in hematologic malignancy patients. Biol Blood Marrow Transplant. (2005) 11(5):389–98. doi: 10.1016/j.bbmt.2005.02.001 15846293

[12] Politikos I Davis E Nhaissi M Wagner JE Brunstein CG Cohen S. Guidelines for cord blood unit selection. Biol Blood Marrow Transplant. (2020) 26(12):2190–6. doi: 10.1016/j.bbmt.2020.07.030 32736011

[13] Brunstein CG Barker JN Weisdorf DJ Defor TE McKenna D Chong SY. Intra-BM injection to enhance engraftment after myeloablative umbilical cord blood transplantation with two partially HLA-matched units. Bone Marrow Transplant. (2009) 43(12):935–40. doi: 10.1038/bmt.2008.417 PMC5157830 19139736

[14] Nishida T Kobayashi T Sawa M Masuda S Shibasaki Y Goto T. A multicenter phase II study of intrabone single-unit cord blood transplantation without antithymocyte globulin. Ann Hematol (2021) 100(3):743–52. doi: 10.1007/s00277-020-04365-z 33427909

[15] Kurita N Gosho M Yokoyama Y Kato T Obara N Sakata-Yanagimoto M. A phase I/II trial of intrabone marrow cord blood transplantation and comparison of the hematological recovery with the Japanese nationwide database. Bone Marrow Transplant. (2017) 52(4):574–9. doi: 10.1038/bmt.2016.319 28067880

[16] Okada M Tasaka T Ikegame K Aotsuka N Kobayashi T Najima Y. A prospective multicenter phase II study of intrabone marrow transplantation of unwashed cord blood using reduced-intensity conditioning. Eur J Haematol (2018) 100(4):335–43. doi: 10.1111/ejh.12999 29168236

[17] Bonifazi F Dan E Labopin M Sessa M Guadagnuolo V Ferioli M. Intrabone transplant provides full stemness of cord blood stem cells with fast hematopoietic recovery and low GVHD rate: results from a prospective study. Bone Marrow Transplant. (2019) 54(5):717–25. doi: 10.1038/s41409-018-0335-x PMC6760547 30232415

[18] Fuchs EJ O’Donnell PV Eapen M Logan B Antin JH Dawson P. Double unrelated umbilical cord blood vs HLA-haploidentical bone marrow transplantation: the BMT CTN 1101 trial. Blood (2021) 137(3):420–8. doi: 10.1182/blood.2020007535 PMC7819761 33475736

[19] Wu KH Tsai C Wu HP Sieber M Peng CT Chao YH. Human application of ex vivo expanded umbilical cord-derived mesenchymal stem cells: enhance hematopoiesis after cord blood transplantation. Cell Transplant. (2013) 22(11):2041–51. doi: 10.3727/096368912X663533 24165586

[20] Sundin M Barrett AJ Ringden O Uzunel M Lonnies H Dackland AL. HSCT recipients have specific tolerance to MSC but not to the MSC donor. J Immunother. (2009) 32(7):755–64. doi: 10.1097/CJI.0b013e3181ab1807 PMC2747778 19561533

[21] Baron F Labopin M Ruggeri A Ehninger G Bonifazi F Stelljes M. Umbilical cord blood versus unrelated donor transplantation in adults with primary refractory or relapsed acute myeloid leukemia: a report from eurocord, the acute leukemia working party and the cord blood committee of the cellular therapy and immunobiology working party of the EBMT. Blood Cancer J (2019) 9(4):46. doi: 10.1038/s41408-019-0204-x 30979868 PMC6461673

[22] Metheny L Politikos I Ballen KK Rezvani AR Milano F Barker JN. Guidelines for adult patient selection and conditioning regimens in cord blood transplant recipients with hematologic malignancies and aplastic anemia. Transplant Cell Ther (2021) 27(4):286–91. doi: 10.1016/j.jtct.2020.11.008 33836867

[23] Le Blanc K Rasmusson I Sundberg B Gotherstrom C Hassan M Uzunel M. Treatment of severe acute graft-versus-host disease with third party haploidentical mesenchymal stem cells. Lancet (2004) 363(9419):1439–41. doi: 10.1016/S0140-6736(04)16104-7 15121408

